# Internal light source for deep photodynamic therapy

**DOI:** 10.1038/s41377-022-00780-1

**Published:** 2022-04-06

**Authors:** Buhong Li, Li Lin

**Affiliations:** MOE Key Laboratory of OptoElectronic Science and Technology for Medicine, Fujian Provincial Key Laboratory for Photonics Technology, Fujian Normal University, Fuzhou, 350117 China

**Keywords:** Lasers, LEDs and light sources, Biomedical materials

## Abstract

Photodynamic therapy (PDT) for deep-seated lesion is seriously hindered by the limited depth of visible light penetration. Most recently, researchers have designed a genetically-encoded NanoLuc-miniSOG with internal light source for self-excitation, which is highly beneficial for deep PDT.

Photodynamic therapy (PDT) is a minimally invasive therapeutic modality, which can selectively destroy diseased tissues by reactive oxygen species (ROS) generated through chemical reaction of photosensitizer (PS) that absorbs irradiation light with an appropriate wavelength in the presence of molecular oxygen. However, the clinical PDT applications are still restricted to superficial and endoscopically accessible lesions since PDT for deep-seated neoplasms is seriously hindered by the limited depth of ultraviolet (UV)-visible (VIS) light penetration^1,2^. The optical penetration depth in tissue is strongly dependent on light wavelength^3,4^. Even for light irradiation at 700–800 nm, the penetration depth is only around 5–6 mm.

As shown in Table 1, different excitation sources have been developed to increase the treatment depth for PDT. Firstly, the direct near-infrared (NIR) light excitation, including two-photon, three-photon, and four-photon absorption, and wavelength conversion with upconversion nanoparticles and nonlinear photon conversion (i.e., coherent anti-Stokes Raman scattering and four-wave mixing) for NIR excitation were used to excite different PSs^5–9^. Secondly, the external excitation sources, such as X-ray, γ-ray and acoustic wave were utilized to excite PS based on direct excitation or resonance energy transfer (RET)^10,11^. Thirdly, the UV-VIS-NIR light with local light delivery systems was performed for interstitial PDT^12,13^. In addition, the internal self-excitation sources, including chemiluminescence, bioluminescence and Cerenkov radiation were investigated to overcome the limited depth of conventional PDT^14–17^. Differing from the first three excitation sources mentioned above, the internal self-excitation source not only avoids the need for an external excitation source, but also makes the limitless treatment depths achievable for PDT^18,19^. Therefore, the internal light source for deep PDT has recently received great attention.

**Table 1:** Excitation sources for deep PDT

Excitation source	Mechanism	Excitation strategies
NIR light	Direct excitation	NIR
		Two-photon
		Three-photon
		Four-photon
	Wavelength conversion	Conjugated with upconversion nanoparticles
		in situ nonlinear photon conversion
External excitation	Direct excitation or RET	X-ray
		γ-ray
		Acoustic wave
UV-VIS-NIR light	Local light delivery	Optical fiber for light delivery
		Implantable and wirelessly powered LED
Internal self-excitation	RET	Chemiluminescence
		Bioluminescence
		Cerenkov radiation

A recent research paper in *Light: Science & Applications*^20^, entitled “Genetically-encoded BRET-activated photodynamic therapy for the treatment of deep-seated tumors”, by Proshkina’s group from Russian Academy of Sciences, reports a proof of concept for the genetically-encoded bioluminescence RET (BRET)-activated PDT^19^. To achieve this, they synthesized genetic NanoLuc-miniSOG BRET pair with the combination of NanoLuc luciferase flashlight and phototoxic flavoprotein miniSOG, and the ROS can be efficiently generated under luciferase substrate injection. The BRET ratio for NanoLuc-miniSOG pair was quantified to be around 0.74 ± 0.05, which indicates high efficiency was obtained for energy transfer in the BRET pair. The BRET-mediated cytotoxicity for the incubated BT-474/NanoLuc-miniSOG cells with membrane localization was obviously observed in the presence of furimazine and Riboflavin mononucleotide (RF), which is correlated with furimazine concentration and reaches 71% at 75 μM furimazine. To further demonstrate BRET-activated PDT in vivo, engineered BT-474 cells stably expressing NanoLuc-miniSOG BRET pair were used to generate tumors in BALB/c nu/nu mice, and the greatest tumor growth inhibition (TGI) of 72% for the BRET- PDT mice was achieved without external light irradiation. More importantly, HER2-specific lentiviruses (LVs) were chosen as the carriers to directly deliver NanoLuc-miniSOG gene into mice bearing tumor. As a result, the mice bearing BT-474 xenograft tumors treated with HER2-specific LV-NanoLuc-miniSOG have the average TGI value of 67%. These obtained results demonstrate that the genetically encoded NanoLuc-miniSOG pair offers a new paradigm for effectively initiating PDT for deep lesions.

The development of the genetically encoded PSs with internal light source provides a less complicated and cost-efficient strategy for efficient BRET-activated PDT, which has great potential for depth-independent PDT and would significantly expand the PDT scope of clinical indications. Moreover, the genetically encoded PSs can be modified with genetic therapy modalities and targeted with specific motifs to particular cellular organelle and cell type, providing the possibility for combination of PDT and immunotherapy.

Despite the genetically encoded PSs with internal light source are rapidly developed in the past decade, there are still several technological challenges need to be thoroughly addressed before clinical translation. First, the ROS quantum yield for miniSOG as PS during photosensitization is extremely low in biological microenvironments^21^, while the cytotoxicity of furimazine and its oxidized product furimamide should be minimized. As a consequence, further improvement should focus on the efficiency of the self-illuminating materials and BRET for higher ROS production. Second, given the physiological complexity of deep-seated neoplasms, more detailed investigations on the biological mechanisms of activation of NanoLuc-miniSOG pair are highly needed. Third, more dosimetric parameters are required to be optimized and standardized for achieving a satisfactory outcome since the treatment efficiency of NanoLuc-miniSOG pair for deep PDT has been complicated by the additional use of furimazine and RF.
